# Supplementary material for: The EBS-A* algorithm: An improved A* algorithm for path planning
Source: PLoS One. 2022 Feb 17;17(2):e0263841. doi: 10.1371/journal.pone.0263841 (PMC8853577; doi:10.1371/journal.pone.0263841)
Supplement: Attachment — Submitted filename: Rebuttal Letter.doc [file pone.0263841.s001.doc]

Dear editor,

We wish to thank you for the time and effort you have spent on reviewing our paper. We are pleased to note that you have found our research work interesting and also pointed out some problems to help us improve the quality of our work.

Motivated by your comments, we have deeply reconsidered the architecture of our work and tried to fix all the problems you mentioned. In particular, this revised manuscript of our resubmitted letter has significantly been improved mainly as follows:

1. Your submission requires editing for English grammar and usage before it could be sent for review.

Response: we purchased the professional scientific editing service from PLOS’ partner with American Journal Experts (AJE) from recommended partner. We have modified the manuscript in English grammar and usage. AJE editing certificate will be submitted to the submission system.

2. We are concerned that the manuscript does not meet PLOS ONE publication criterion number 3, which states that experiments, statistics, and other analyses are performed to a high technical standard and are described in sufficient detail. We are concerned that several details are unclear in the manuscript, including details in the Methods section of the hardware used and how the physical experiments were carried out. Without these details, we do not feel that the study is reproducible by another researcher.

Response: Regarding this comment, we have supplemented the description of the mobile robot hardware platform and the basic steps of the physical experiments in the manuscript. In addition, we shared the experimental source codes and detailed experimental procedures on GitHub for this study is reproduced by another researcher. The GitHub link: <https://github.com/wanghw1003/EBAStar>.

We deeply appreciate your consideration of our manuscript, and we look forward to receiving comments from the reviewers.

Thank you and best regards.

Yours sincerely,

Huanwei Wang
