# Supplementary material for: The EBS-A* algorithm: An improved A* algorithm for path planning
Source: PLoS One. 2022 Feb 17;17(2):e0263841. doi: 10.1371/journal.pone.0263841 (PMC8853577; doi:10.1371/journal.pone.0263841)
Supplement: Attachment — Submitted filename: Response to Reviewers.docx [file pone.0263841.s002.docx]

Dear reviewer,

Thank you for your valuable comments and suggestions concerning our manuscript. The comments are very helpful for revising and improving the paper, as well as the important guiding significance to our researches. We have studied comments carefully and have made corrections. We hope you find the revised manuscript acceptable for publication. Below, we have provided answers to the questions raised by the reviewer, along with detailed explanations for your concerns.

**Response to Reviewer 1 Comments**

**Point 1:** In section 2 (Related work), when explaining the related literature and the proposed A*-based algorithms' difficulties, please, mention how your proposed method handles those difficulties and contributes to the corresponding literature.

**Response 1:**

we have completely rewritten the Related work section and reorganized and analyzed the references, it mainly focuses on the efficiency and robustness of the algorithm. Firstly, we analyzed several improved A* algorithms and these variants in efficiency and summarized the existing shortcomings. Secondly, we analyze several literatures to enhance the reliability of the algorithm and some methods, such as smoothing, and summarize the shortcomings of these improved algorithms. We found that the performance of the existing A* improved algorithm is not balanced, and lacks the A* improved algorithm with high efficiency and strong robustness. Based on the analysis and summary of the references, we propose a combined improvement strategy of the A* algorithm.

**Point 2:** How the expansion distance is automatically decided for different environments? Is it only one extra grid cell from the sides of the obstacle or it can include multiple layers of grid cells on the sides of the obstacle? how does the algorithm decide to choose the width of the expansion distance in accordance with the environmental scale?

**Response 2:**

It is not only one extra grid cell from the sides of the obstacle, multiple layers of grid cells can be included on the sides of the obstacle. Regarding the question of how many layers of grid cells extend around the obstacle, this appropriate distance is related to the robot size, the map scale, and the traveling speed of the robot. How to automatically decide the expansion distance in different environments? We simplified and equivalent to a model, and made a simplified model of the robot and obstacles. Based on these constraints, we constructed the mathematical relationship between the expansion distance and the cruising speed of the robot. Based on this knowledge, the expansion distance can be decided automatically and reasonably.

**Point 3:** Can Fig.2 be modified to see how the proposed bidirectional search handles the situation when there is an obstacle inside the grids?

**Response 3:**

We have added two graphs, respectively, when there is one obstacle and multiple obstacles inside the grids. Through these graphs, we can clearly understand how the bidirectional search handles the situations when encountering obstacles.

**Point 4:** On page 16, " and the number is 1% of the map scale", is not clear.

**Response 4:**

We have revised the unclear words.

After revised: The location of obstacles is randomly generated on the map based on the center point, but there are certain rules. The scale of the obstacle occupies a certain proportion of the map scale, which is interpreted as the number of obstacle center points is 1% of the map scale.

**Point 5:** I would like to see 4 sets of simulation tests similar to Fig. 5 (including it) with different map scales (100x100, 150x150x, 200x200x) and a different number of obstacles (demonstrating less cluttered to highly cluttered environments) to confirm the performance of the proposed method.

**Response 5:**

We have added three groups of simulation tests on different map scales (100x100, 150x150x, and 200x200), including the tests of fixed maps and the multiple tests of randomized maps. Set up obstacles of different densities on maps of different scales to represent from less cluttered to highly cluttered environments. We have analyzed the experimental results, and the algorithm has shown results in line with expectations on different scale maps.

**Response to Reviewer 2 Comments**

**Point 1:** For related work section, you should discuss the algorithms based on their classification either classical or meta-heuristic or other. Then summarize your findings.

**Response 1:**

we have completely rewritten the Related work section and rediscussed the classification of path planning algorithms. We reorganized and analyzed the references, focusing on the efficiency and robustness of the algorithm, and summarized the existing shortcomings of improved A* algorithms and these variants. We found that the performance of the existing improved A* algorithms is not balanced, and lacks the improved A* algorithm with high efficiency and strong robustness. Based on the analysis and summary of the literatures, we propose a combined improvement strategy of the A* algorithm.

**Point 2:** There is no proper justification on why A* is improved rather than Artificial Potential Field or D* where have better performance compare to A* in some literatures.

**Response 2:**

The application scenario of this research is path planning in static planning (or global path planning), and the A* algorithm is one of the most well-known and most widely used algorithms in static path planning. The Artificial Potential Field and D* are path planning algorithms used in dynamic planning scenarios. The revised content of the manuscript is as follows:

Path planning algorithms are also divided into global path planning and local path planning based upon available environmental knowledge. Global path planning seeks an optimal path given largely complete environmental information and is best performed when the environment is static and perfectly known to the robot. Therefore, global path planning is also called static path planning. By contrast, local path planning is most typically performed in unknown or dynamic environments, local path planning is also called dynamic path planning.

For application scenarios such as warehousing and logistics, path planning in a static environment assumes that the robot perceives the environment and uses local path planning algorithms when the environmental information is not fully grasped. A* is used for shortest path evaluation based on the information regarding the obstacles present in the static environment[10], and the shortest path evaluation for the known static environment is a two-level problem, which comprises a selection of feasible node pairs and shortest path evaluation based on the obtained feasible node pairs[11]. Both of the above-mentioned criteria are not available in a dynamic environment, which makes the algorithm inefficient and impractical in dynamic environments. In this case, the dynamic path planning algorithm is not suitable for use in a static environment. Classical algorithms include D* algorithm, Artificial Potential Field algorithm, and etc. A* algorithm is chosen because it represents the foundational algorithms used within contemporary real-time path planning solutions in a static environment. Novel research builds on the algorithm to find additional performance and efficiency.

**Point 3:** The pseudocode format is incorrect. Please correct them.

**Response3:**

We have revised the pseudocode format to keep it in the correct format.

**Point 4:** Why Expansion distance and Bidirectional search optimization are considered to be hybridized with A*? Please justify them with proper support.

**Response 4:**

We have added the theory of expansion distance and Bidirectional search in this manuscript to support the rationality of the proposed optimization strategy. Since A* algorithm is a path planning algorithm based on graph search and evolved from BFS, therefore, it is reasonable to apply two optimization strategies based on graph node operation to improve the A* algorithm.

**Point 5:** I would like to suggest at least three scenarios for simulation test (no obstacle, several obstacles, and a lot of obstacles).

**Response 5:**

We have added three groups of experiments on different map scales (100*100, 150*150, 200*200). Like the experiments in the manuscript, we performed the tests in fixed maps and randomized maps. Set up obstacles of different densities on maps of different scales to represent from less cluttered to highly cluttered environments. However, we did not add the tests in no obstacle scenarios, our considerations are as follows:

The difference between the EBS-A* algorithm and the traditional A* algorithm is that the efficiency of the algorithm is improved by expanding the distance and bidirectional search, and the robustness of the algorithm is enhanced by expanding the distance and smoothing. An environment without obstacles is a collision-free environment, if the robot will not collide during the travel, the robustness of the algorithm will not be affected, and the advantage of the EBS-A* algorithm will no longer be obvious. In a barrier-free environment, there is no fundamental difference between the paths planned by the two algorithms, and the efficiency of bidirectional search will be twice that of single search, and the algorithm will strictly follow this rule.

**Point 6:** Comparison graphs to compare the performance of the algorithms proposed against the existing one.

**Response 6:**

We have added the comparison graph to compare the performance of the algorithms proposed against the existing one. The graph is as follow:

**Point 7:** There is no significant analysis done by the authors. Please consider them.

**Response 7:**

We adjusted the chapter structure, added the discussion section, moved the part that involved analysis and discussion in the original simulation test section to discussion section. We divide the discussion into two sections, namely efficiency and robustness, to discuss separately. The fixed maps, randomized maps, and comparative experiments were discussed and analyzed in turn. It also gives a general analysis of the algorithm in the environment of different density obstacles.

**Point8:** The effectiveness test only utilize EBS-A* algorithm. Why not other existing algorithm as well?

**Response 8:**

The effectiveness test is to verify the effectiveness and feasibility of the proposed algorithm in mobile robots and real physical scenarios and to verify whether the algorithm has the potential for industrial applications. Simulation test is only a way to verify the performance of the algorithm, and the real hardware platform test is to more comprehensively verify the application value of the algorithm and the potential of industrial applications. After the reviewers’ comment and in-depth consideration, the section called the Effectiveness test is indeed unreasonable, so it is called a Real-world case. We have modified this section as a Real-world case and made slight changes to the content.

**Point 9:** Please proof read your manuscript before your resubmission.

**Response 9:**

We adopted the reviewer's comments and carefully proofread the full text of the manuscript based on extensive revisions.
