# Supplementary material for: The EBS-A* algorithm: An improved A* algorithm for path planning
Source: PLoS One. 2022 Feb 17;17(2):e0263841. doi: 10.1371/journal.pone.0263841 (PMC8853577; doi:10.1371/journal.pone.0263841)
Supplement: Attachment — Submitted filename: Response to Reviewers.docx [file pone.0263841.s003.docx]

Dear reviewer,

Thank you for your valuable comments and suggestions concerning our manuscript. The comments are very helpful for revising and improving the paper, as well as the important guiding significance to our researches. We have studied comments carefully and have made corrections. We hope you find the revised manuscript acceptable for publication. Below, we have provided answers to the questions raised by the reviewer, along with detailed explanations for your concerns.

**Response to Reviewer 1 Comments**

**Point 1:** The English of the manuscript still requires improvement.

**Response 1:**

We have read the full text of the manuscript and revised some English issues.

**Point 2:** Line 48, and line 53 need correction.

**Response 2:**

We have revised the two incorrect sentences.

Line 48: In section 7, the EBS-A* algorithm is transplanted to a robot and verified the effectiveness of the algorithm is verified in the real world.

**Revise**: In section 7, the EBS-A* algorithm is tested in the robot operating system (ROS) and its performance is verified in the real world.

Line 53: Regarding the classification of the path planning algorithms, path planning algorithms are divided into more than two groups.

**Revise**: There are multiple classification methods for path planning algorithms.

**Point 3:** Line 60-61, what do those abbreviations stand for?

**Response 3:**

GA represents the genetic algorithm, ACO algorithm stands for ant colony optimization algorithm. These two abbreviations are first mentioned in lines 12-13. ANN algorithm is artificial neural network algorithm, SA algorithm is simulated annealing algorithm, all issues have been revised.

**Point 4:** Very long sentence from line 71 to 75.

**Response 4:**

We have revised the long sentence.

**Revise:** A* is used for shortest path evaluation based on the information regarding the obstacles present in the static environment. The shortest path evaluation for the known static environment is a two-level problem. The problem comprises a selection of feasible node pairs and the shortest path evaluation based on the obtained feasible node pairs.

**Point 5:** the paragraph starting from line 128 and its intentions are unclear.

**Response 5:**

We have revised the unclear sentence in the manuscript.

Line 128: A constrained A* method is proposed for USVs in a maritime environment.

**Revise**: A constrained A* method is proposed for unmanned surface vehicles (USVs) in a maritime environment.

**Point 6:** the paragraph starting from line 152 is missing a strong motivation for this study.

**Response 6:**

We have added the motivation for this study.

Revise: The existing improved A* algorithm only optimizes efficiency or robustness. At present, there is no improved algorithm with excellent comprehensive performance. Path planning algorithm plays an essential role in the autonomous navigation of mobile robots. Since mobile robots are widely used in the real world, it is very necessary to propose an improved A* algorithm with strong robustness and high efficiency. It has huge application potential and commercial value in the industrial field.

**Point 7:** line 156 "The A* algorithm was first proposed and described in detail in [13]," should be double fact-checked. A* has been proposed firstly half a century ago.

**Response 7:**

Following your suggestion. We have revised this reference in the manuscript.

**Point 8:** Line 175 needs correction.

**Response 8:**

We have corrected the sentence.

In this section, three optimization strategies are proposed to improve the efficiency and robustness of the A* algorithm.

**We responded to points 9-14 together.**

**Point 9:** In formula (2), the sub index of E(V__i_) should be corrected, i.e., should be without underscore. The * (if denotes multiplication) is redundant and should be removed. Throughout the paper, often the text format of parameters in formulas and their descriptions in the text do not match.

**Point 10:** The assumption in line 216 does not make sense. Assuming that the side length of the square is equal to the radius of the robot is a very poor assumption since the obstacles can vary in size greatly. Assuming all obstacles are identical in size with the robot is not realistic.

**Point 11:** In formula (2), V_i_ is introduced as the current cruising speed of the robot and V_r_ is the cruising speed of the robot. What is the difference between these two speeds? It looks likes they refer to an identical speed.

**Point 12:** In formula (2), E(V_i_) is introduced as the number of expansion nodes. Does this mean how many nodes will be considered around an obstacle? Wasn't the main question determining the side length of an expansion node rather than their number?

**Point 13:** In formula (2), no need to use the word "default".

**Point 14:** Considering my comments above about formula (2), this formula should be redefined and show more clearness.

**Response:**

We have revised all the problems in point 9-14, the specific content is as follows:

How is the expansion distance automatically decided for different environments? This is a question we must consider. We refer to the robot equivalent model in the robot operating system to discuss the expansion distance. In this manuscript, the following assumptions of the robot and obstacles are made to simplify the model.

The robot model is equivalent to a cylinder in ROS, with a radius of $r$ and cruising speed $V_{r}$, which satisfies $V_{r}\leq V_{max}$, where $V_{max}$ is the maximum cruising speed determined by the performance of the robot. $V_{r}$ is a speed threshold, $V_{i}$ is the current speed of the robot. When $V_{i}\leq V_{r}$, the expansion distance is only expanded by one node, and when the current speed is greater than $V_{r}$, the probability of a collision between the robot and an obstacle increases. In this case, the number of expanded nodes should increase. The obstacle is equivalent to one grid or more square grids. The mapping rule between the robot model and the map is that the robot radius is equal to the length of a grid. The mapping rule between the number of expansion nodes and the speed is that the ratio of the current speed and the speed threshold.

$$E\left( V_{i} \right)=\left\{ \begin{aligned} r V_{i}\leq V_{r} \\ \frac{V_{i}}{V_{r}}r V_{r}<V_{i}\leq V_{max} \end{aligned} \right.$$

where $E\left( V_{i} \right)$ is the number of expansion nodes. Regarding the selection of the expansion distance magnitude, when $V_{i}\leq V_{r}$, the expansion distance defaults to the radius of the cylinder as the expansion distance. This distance provides a sufficient “collision buffer” for the reliability of the path, and ensure acceptably minimal waste of the physical space the robot travels. The bilateral expansion distances are the size of a robot itself when there are obstacles on both sides. As the speed of the robot increases, the risk of robot collisions will increase. Correspondingly, only expanding the expansion distance can ensure that the robustness of the algorithm does not decrease. When $V_{i}$ increases, $E\left( V_{i} \right)$ should increase accordingly, otherwise the risk of robot collision will increase. Therefore, the determination of the expansion distance follows a linear relationship with the speed $V_{i}$.

**Point 15:** Figure 2 is unclear.

**Response 15:**

We have redrawn Figure 2.

**Point 16:** Algorithm one still is not clearly explaining the idea. Maybe a flowchart can be a better representation.

**Response 16:**

We have added a flowchart of the algorithm and the explanation.

The execution process of the EBS-A* algorithm consists of four steps: 1. Performing expansion distance optimization on the A* algorithm. 2. Performing bidirectional search on the algorithm. 3. Generating an unsmooth path. 4. Performing smoothing process to generate a smooth path. The execution process of the proposed algorithm is shown in Fig 6.

**Point 17:** The experimental results (figures and discussions) of autonomous navigation testing are missing. How the experiment robot has found its collision-free path toward the goal location using the proposed algorithm?

**Response 17:**

We have added the experimental results and the experiment process.

The start node and the end node are set in the real world. The robot uses the EBS-A* algorithm to plan a collision-free path to the goal position. This experiment process consists of three steps:

1. EBS-A* algorithm transplantation;

2. simultaneous localization and mapping (SLAM) test;

3. a robot autonomous navigation test.

Algorithm transplantation accomplishes writing EBS-A* algorithm into the ROS. The SLAM test built a test map to use the radar on the robot in the real world. The autonomous navigation test verifies the effectiveness of the algorithm in the real world.

In this experiment, we set an application environment for EBS-A* algorithm and carried out the autonomous navigation test. The EBS-A* algorithm was written into a real mobile robot. The experimental result shows that the robot can independently plan a reliable and smooth path and complete the autonomous navigation from the starting node to the goal node. This experiment verifies that the EBS-A* algorithm can be applied to mobile robots and has the potential to be applied to industrial scenarios.

**Point 18:** Figure 12 does not completely match Table 9.

**Response 18:**

We corrected the data in Table 9 and added the explanation to Table 9 and the original Figure 12 (now Figure 13).

In this research, we have tested the efficiency of the traditional A* algorithm and EBS-A* algorithm. In [16], authors also have tested the efficiency of the traditional A* algorithm and the geometric A* algorithm. We can choose the efficiency of the traditional A* algorithm as a benchmark to compare the efficiency of the EBS-A* algorithm and the geometric A* algorithm. As shown in Table 9, the running time of the A* algorithm is 316.334s and the running time of the geometric A* algorithm is 292.142s in [16]. The running time of the traditional A* algorithm is 36.806s and the running time of the EBS-A* algorithm is 9.747s. The histograms of EBS-A* and geometric A* in Fig 13 are the results of proportional calculations.

**Point 19:** The theoretical part of the work must be developed significantly.

**Response 19:**

We have added basic theoretical support to the bidirectional search and smoothing part of the manuscript.

**Bidirectional search:**

The traditional graph search algorithms are not considered the features of the path planning problem, like DFS and BFS. The path is searched by the strategy set beforehand for any problem, and the search process will not be optimized according to the features of the problem. The A* algorithm is developed based on the BFS algorithm. The concept of heuristic is introduced on the basis of the BFS algorithm. The heuristic information is obtained according to the features of the problem, which will guide the search in the optimal direction. Such as speeding up the search process and improving efficiency. The traditional A* algorithm uses Manhattan distance as its heuristic equation. Manhattan distance is defined as follows:

$h\left( n \right)=\left| x_{a}-x_{b} \right|+\left| y_{a}-y_{b} \right|$	(3)

**Smoothing:**

Bezier curve is a space curve and has good geometric properties, which is proposed by the French engineer Pierre Bezier in 1962. Bezier curve is one of the methods used to smooth the path, it has been widely used in computer graphics and computer-aided design. If the control point of the Bezier curve is a convex polygon, that is, the feature polygon is convex, the Bezier curve is also convex, which is one of the advantages of the Bezier curve. Unlike other types of curves, such as cubic splines or polynomials, Bezier curve does not pass through all the data points used to define it. The points used to define Bezier curve are called control points. Polygons that can be drawn from these control points are called Bezier polygons. The turning points are the points where the slope of the curve changes its sign. Bezier curves have fewer turning points so that it is smoother than cubic splines.

Bezier curve has the following properties:

1. Symmetry, the $i^{th}$ coefficient of the curve is the same as the reciprocal $i^{th}$ coefficient.

2. Convex hull properties, Bezier curve is always contained in the convex hull of the polygon defined by all control points.

3. End-points properties, the first control point and the last control point on the curve are exactly the start point and the end point of the Bezier curve.

4. Recursion, which means that the coefficient of the Bezier curve satisfies the following formula.

$B_{i,n}\left( t \right)=\left( 1-t \right)B_{i,n-1}\left( t \right)+tB_{i-1,n-1}\left( t \right),\left\{ i=0,1,\cdots,n \right\}$	(4)

The radius of curvature of the Bezier curve varies smoothly from the starting point to the endpoint because of its continuous higher order derivatives. A Bezier curve of degree n is a parametric curve composed of Bernstein basis polynomials of degree $n$ and it can be defined as:

$P\left( t \right)=\sum_{i=1}^{n} p_{i}B_{i,n}\left( t \right),t\in\left[ 0,1 \right]$	(5)

Where $t$ indicates the normalized time variable, $P_{i}{(x_{i},y_{i})}^{T}$ represents the coordinate vector of the $i^{th}$ control point with $x_{i}$ and $y_{i}$ being the components corresponding to the X and Y coordinate, respectively, $B_{i,n}$ is the Bernestein basis polynomials, which represents the base function in the expression of Bezier curve, and it is defined as follows:

$B_{i,n}\left( t \right)=C_{n}^{i}t^{i}=\frac{n!}{i!(n-i)!}t^{i}{(1-t)}^{n-i},i=0,1,\cdots n.$	(6)

The derivatives of Bezier curve are determined by the control points, and the first derivative of a Bezier curve in formula 5. is expressed as in formula 7. Moreover, higher-order derivatives of a Bezier curve can also be calculated.

$\dot{P\left( t \right)=\frac{dP(t)}{dt}}=n\sum_{i=0}^{n-1} B_{i,n-1}\left( t \right)(P_{i+1}-P_{i})$	(7)

In the two-dimensional space, the curvature of a Bezier curve with respect to t is expressed as follows:

$k\left( t \right)=\frac{1}{R(t)}=\frac{\dot{P}_{x}\left( t \right)\ddot{P}_{y}\left( t \right)-\dot{P}_{y}(t)\ddot{P}_{x}(t)}{{(\dot{P}_{x}^{2}\left( t \right)+\dot{P}_{y}^{2}(t))}^{1.5}}$	(8)

In the path planning problem, Bezier curve is connected to form a smooth path planning for mobile robots.

**Response to Reviewer 2 Comments**

**Point 1:** I would suggest the authors to add some statistical analysis on the results obtained.

**Response 1:**

We have added some statistical analysis in this manuscript.

As shown in Table 3，the running time of the EBS-A* algorithm is 138.813s and the traditional A* algorithm is 302.467s on a 100×100 map. The efficiency of the EBS-A* algorithm is only 2.17 times that of the A* algorithm. As shown in Table 4, the efficiency of the EBS-A* algorithm is only 2.14 times that of the A* algorithm on randomized 100×100 maps. The statistical results in Table 3 and Table 4 show that the test results of the algorithm on the fixed map and the random map are consistent. The efficiency of the algorithm is reliable.

But the efficiency of the EBS-A* algorithm is 4.7 times that of the A* algorithm on a 150×150 map. The reason is that there are only a few obstacles in the 100×100 map, but there are dense obstacles in the 150×150 map. In an environment with dense obstacles, the efficiency of the algorithm is higher. These statistical results have also been verified on 200×200 maps. the efficiency of the EBS-A* algorithm is 5.79 times that of the A* algorithm on a 200×200 map.

All the statistical results of the simulation test show that the efficiency of the EBS-A* algorithm is significantly improved compared with the traditional A* algorithm. These results verify the rationality of the algorithm design.
