# Supplementary material for: The EBS-A* algorithm: An improved A* algorithm for path planning
Source: PLoS One. 2022 Feb 17;17(2):e0263841. doi: 10.1371/journal.pone.0263841 (PMC8853577; doi:10.1371/journal.pone.0263841)
Supplement: Attachment — Submitted filename: Response to Reviewers.docx [file pone.0263841.s004.docx]

Dear reviewer,

Thank you for your valuable comments and suggestions concerning our manuscript. The comments are very helpful for revising and improving the paper, as well as the important guiding significance to our researches. We have studied comments carefully and have made corrections. We hope you find the revised manuscript acceptable for publication. Below, we have provided answers to the questions raised by the reviewer, along with detailed explanations for your concerns.

**Response to Reviewer 1 Comments**

**Point 1:** Line 279, what denote xa,xb,ya, and yb? it should be given after the formula.

**Response 1:**

$h\left( n \right)=\left| x_{a}-x_{b} \right|+\left| y_{a}-y_{b} \right|$	(3)

We have added the statements in this manuscript.

The heuristic function is the minimum cost evaluation value of the A* algorithm from any node to the goal node and helps to reduce the number of nodes traversed. The heuristic function of the traditional A* algorithm uses the Manhattan distance.

In formula (3), $h(n)$ is the heuristic function, $(x_{a},y_{a})$ is the coordinates of the goal node, $(x_{b},y_{b})$ is the coordinates of any node.

In addition, we have checked the full text of the manuscript to ensure that there are no such issues.

**Point 2:** Starting from line 289, the Bezier curve theory is explained. However, this method, if used to smooth the path, is not applied in Fig. 4 and Fig. 5. Also, the Bezier curve should be derived for a "single-angle turn" (Fig. 4) and "continuous right-angle turns" (Fig. 5) for the n right-angle turn case.

**Response 2:**

We have replaced the Fig. 4 and Fig. 5.

They are as follows:

Fig. 4 Smoothing optimization strategy for a single right-angle turn

Fig. 5 Smoothing optimization strategy for continuous right-angle turns

In Fig. 4, we first decompose a single right-angle turn into two acute angles, and two ${45}^{^{\circ}}$angles are generated in the path, as shown in the right picture of Fig. 4 in the original manuscript. We perform Bezier curve smoothing on the ${45}^{^{\circ}}$ angles, which ultimately generates the right picture of Fig. 4. The smoothing process of Fig. 5 is similar to that of Fig.4.

The Bezier curve for a single right-angle turn is a second-order Bezier curve, its formula is:

$$B\left( t \right)={(1-t)}^{2}P_{0}+2t\left( 1-t \right)P_{1}+t^{2}P_{2},t\in\left[ 0,1 \right]$$

$P_{0}$ is the start node, $P_{1}$ is the control node, $P_{2}$ is the end node, and $t$ is time.

A line segment described by a continuous point $Q_{0}$ from $P_{0}$ to $P_{1}$. A line segment is described by a continuous point $Q_{1}$ from $P_{1}$ to $P_{2}$. The continuous node $B(t)$ from $Q_{0}$ to $Q_{1}$ describes a second-order Bezier curve.

The Bezier curve for continuous right-angle turns is a third-order Bezier curve, its formula is:

$$B\left( t \right)={(1-t)}^{3}P_{0}+3t{(1-t)}^{2}P_{1}+ 3t^{2}(1-t)P_{2}+ t^{3}P_{3},t\in\left[ 0,1 \right]$$

$P_{0}$ is the start node, $P_{1}$ and $P_{2}$ are the control nodes, and $P_{3}$ is the end node. The curve passes through the control nodes at both ends, namely:

$$P\left( 0 \right)=P_{0}$$

$$P\left( 1 \right)=P_{3}P^{'}\left( 0 \right)=3(P_{1}-P_{0})$$

**Point 3:** The paths generated by the proposed algorithm do not show the Bezier curve's promised smoothness, for example in Fig. 7.

**Response 3:**

In this manuscript, we apply the proposed algorithm to perform simulation tests on 50×50 maps, 100×100 maps, 150×150 maps and 200×200 maps. In these maps, each grid represents a pixel. The resolution of the test maps is relatively low. Each pixel is a square. The process of path planning is based on pixel points, and the paths generated by the proposed algorithm are composed of pixel points, therefore, the smoothness promised by Bezier curve is not shown in the above maps. In this manuscript, both the theoretical analysis and the real-world case demonstrate the excellent smoothness of the proposed algorithm, as shown in Fig. 16.

**We have made all data underlying the findings in our manuscript fully available on GitHub.**
